# Supplementary material for: Rare diseases load through the study of a regional population
Source: PLoS Genet. 2025 Oct 16;21(10):e1011876. doi: 10.1371/journal.pgen.1011876 (PMC12530595; doi:10.1371/journal.pgen.1011876)
Supplement: S1 Text — Table A: Variants previously described in the population not found here. Table B: Experimental assessment of CR in an independent cohort of 1,000 individuals living in SLSJ. CI have been calculated on proportions using the online tool https://sample-size.net/confidence-interval-proportion/; * CR calculated using WGS in the present study; ** One sided 97.5% CI. Table C: Case reports on Quebec founder variants. Table D: Comparison of imputations of ClinVar rare variants using the TOPMed r2 or the Quebec reference panel. Proportions are based on common SNPs/genotypes except for missing SNPs which is on the total number of SNPs. False positives are defined as heterozygotes in imputed, but not in WGS data. False negatives are defined as heterozygotes in WGS, but not in imputed data. * In at least one individual. **SNPs having at least one homozygote switch genotype in addition to SNPs having more than 10% of false positive genotypes (101 out of the 106 false positive SNPs) were considered as unreliable in the imputed data. Fig A: Comparison of the variants’ carrier rates reported in SLSJ and found in our analysis. When available, the aggregated CR (all variants associated with the same disease) was used; also, if available, the CR from the imputed data was used; otherwise, the CR from WGS data was utilized. Variants from the same disease were grouped as in previous studies. Fig B: UMAP of WGS data. UMAP are coloured according to A) the recruitment region or country of birth and B) the k-means clustering. Fig C: UMAP of imputed data. UMAP are coloured according to A) the recruitment region or continent of birth and B) the k-means clustering. Note that 1,537 pathogenic variants had an RFD ≥ 10% in the WGS data, but only 1,302 of them had also an RFD ≥ 10% in the imputed data. Fig D: Correlation between imputed and WGS variants’ frequency in QcP. (PDF) [file pgen.1011876.s002.pdf]

# S1 Text
# Rare diseases load through the study of a regional population

### Table A: Variants previously described in the population not found here.

| Nucleotide | Gene | Disease name (ClinVar ID) | Position GRCh38 | Reason | Reference |
|---|---|---|---|---|---|
| **Variants not found in this study** | | | | | |
| Deletion of exons 3–10 | *CTNS* | Cystinosis (4445) | chr17:3600934-3658165del | Variant not found in our data | Laberge, 2005(1) |
| c.397A>T | *CTNS* | Cystinosis (267307) | chr17:3655288:A:T | | Laberge, 2005(1) |
| c.1141A>G | *FAH* | Tyrosinemia type I (11872) | chr15:80181120:A:G | | Scriver, 2001(2) |
| c.1584_1585delinsG | *SLC12A6* | Agenesis of the corpus callosum with peripheral neuropathy (136175) | chr15:34250637-34250638delins | | Bchetnia, 2021(3) |
| c.7504C>T | *SACS* | Charlevoix-Saguenay spastic ataxia (5513) | chr13:23336372:C:T | | Bchetnia, 2021(3) |
| c.2000G>A | *LDLR* | Hypercholesterolemia, familial, 1 (3689) | chr19:11120382:G:A | | Bchetnia, 2021(3) |
| del 15kb | *LDLR* | Hypercholesterolemia, familial, 1 (3709) | chr19:11077742del | | Bchetnia, 2021(3) |
| c.1467C>G | *LDLR* | Hypercholesterolemia, familial, 1 (161270) | chr19:11113643:C:G | | Bchetnia, 2021(3) |
| c.682G>A | *LDLR* | Hypercholesterolemia, familial, 1 (3691) | chr19:11105588:G:A | | Laberge, 2005(1) |
| c.74C>T | *KRT5* | Epidermolysis bullosa simplex (14648) | chr12:52520223:C:T | | Bchetnia, 2021(3) |
| c.449T>C | *KRT5* | Epidermolysis bullosa simplex (66241) | chr12:52519848:T:C | | Bchetnia, 2021(3) |
| c.1130T>C | *KRT5* | Epidermolysis bullosa simplex (66303) | chr12:41583379:T:C | | Bchetnia, 2021(3) |
| c.6275_6276del | *BRCA2* | Familial cancer of breast (9318) | chr13:32340630del | | Laberge, 2005(1) |
| c.6085G>T | *BRCA2* | Familial cancer of breast (52008) | chr13:32340440:G:T | | Laberge, 2005(1) |
| c.2588dup | *BRCA2* | Familial cancer of breast (37793) | chr13:32336936dup | | Laberge, 2005(1) |
| del7.6kb | *HEXA* | Tay-Sachs disease (3891) | chr15:72370592-72378536del | | Laberge, 2005(1) |
| c.889C>T | *PAH* | Phenylketonuria (102885) | chr12:102851710:C:T | | Laberge, 2005(1) |
| c.*224CTG[758] | *DMPK* | Steinert myotonic dystrophy syndrome (188028) | chr19:45770204dup | | Laberge, 2005(1) |
| c.1222C>T | *PAH* | Phenylketonuria (577) | chr12:102840493:C:T | | Laberge, 2005(1) |
| c.93-21G>A | *HBB* | beta Thalassemia (15454) | chr11:5226820:G:A | | Laberge, 2005(1) |
| g.6725GAA[(200_900)] | *FXN* | Friedreich ataxia with retained reflexes (561195) | chr9:69037287dup | | Laberge, 2005(1) |
| c.3649_3650insA | *BRCA1* | Breast-ovarian cancer, familial, susceptibility to, 1 (125644) | chr17:43091881ins | | Laberge, 2005(1) |
| c.2834_2836delinsC | *BRCA1* | Breast-ovarian cancer, familial, susceptibility to, 1 (54694) | chr17:41244712delins | | Laberge, 2005(1) |
| c.1069G>T | *FAH* | Tyrosinemia type I (11871) | chr15:80181048:G:T | | Scriver, 2001(2) |
| c.3GGC[10] | *PABPN1* | Oculopharyngeal muscular dystrophy (279930) | chr14:23321471dup | | Laberge, 2005(1) |
| c.918_919insA | *WNK1* | Charcot-Marie-Tooth disease (637898) | chr12:813800ins | | Laberge, 2005(1) |
| c.194T>C | *PAH* | Phenylketonuria (636) | chr12:102894893:T:C | Variant not with RFD≥10% | Laberge, 2005(1) |
| c.782G>A | *PAH* | Phenylketonuria (582) | chr12:102852875:G:A | | Scriver, 2001(2) |
| c.118C>T | *HBB* | beta Thalassemia (15402) | chr11:5226774:C:T | | Laberge, 2005(1) |
| c.845G>A | *HFE* | Hemochromatosis type 1 (9) | chr6:26092913:G:A | | Laberge, 2005(1) |
| **Variants not considered in this study** | | | | | |
| c.6594delT | *SACS* | Charlevoix-Saguenay spastic ataxia (5512) | chr13:23335032del | Variant duplicate | Laberge, 2005(1) |
| c.5254C>T | *SACS* | Charlevoix-Saguenay spastic ataxia (5513) | chr13:23336372:C:T | | Laberge, 2005(1) |

| | | | | | |
|---|---|---|---|---|---|
| c.3897T>G | *LRPPRC* | Leigh syndrome, French-Canadian type /Congenital lactic acidosis, Saguenay-Lac-Saint-Jean type (3110) | chr2:43974244:T:G | | Laberge, 2005(1) |
| c.1119C>T | *LRPPRC* | Leigh syndrome, French-Canadian type /Congenital lactic acidosis, Saguenay-Lac-Saint-Jean type (3110) | chr2:43974244:C:T | | Laberge, 2005(1) |
| 621+1G>T | *CFTR* | Cystic fibrosis (38799) | chr7:117531115:G:T | | Bchetnia, 2021(3) |
| c.187C>G | *HFE* | Hemochromatosis type 1 (10) | chr6:26090951:C:G | Too frequent | Laberge, 2005(1) |
| No variant | *PKLR* | Pyruvate kinase deficiency (NA) | NA | Variant without any position | Cruz Marino, 2023(4) |
| c.958delG | *CYP27B1* | Vitamin D-dependent rickets, type 1A (NA) | NA | | Bchetnia, 2021(3) |
| No variant | *SARDH* | Sarcosinemia (NA) | NA | | Cruz Marino, 2023(4) |
| No variant | *HAL* | Histidinemia (NA) | NA | | Cruz Marino, 2023(4) |
| del5kb | *LDLR* | Hypercholesterolemia, familial, 1 (NA) | NA | | Laberge, 2005(1) |
| Apo E2 allele | *APOE* | Familial dysbetalipoproteinemia (type III hyperlipoproteinemia) (NA) | NA | | Bchetnia, 2021(3) |
| nt1035incC | *CTNS* | Cystinosis (NA) | NA | | Laberge, 2005(1) |
| c.-128GGM[55_?] | *FMR1* | Fragile X syndrome (9972) | NA | X chromosome | Laberge, 2005(1) |
| No variant | *S26W* | X-linked hereditary neuropathy (NA) | NA | | Laberge, 2005(1) |

**Table B: Experimental assessment of CR in an independent cohort of 1,000 individuals living in SLSJ.**

| Gene | Nucleotide | This study CR | Reassessed CR | Lower CI (95%) | Upper CI (95%) |
|---|---|---|---|---|---|
| DOK7 | c.1124_1127dup | 1/21 | 1/29 | 1/42 | 1/21 |
| CTNS | c.414G>A | 1/63* | 1/43 | 1/68 | 1/29 |
| CC2D2A | c.4667A>T | 1/33 | 1/66 | 1/118 | 1/40 |
| SGO1 | c.67A>G | 1/51 | 1/83 | 1/159 | 1/47 |
| CTNS | c.473T>C | 1/513 | 1/990 | 0 | 1/179 |
| GALNS | c.1171A>G | 1/276 | 0/990 | 0 | 1/270** |
| ETFA | c.495_496del | 1/103 | 1/196 | 1/588 | 1/84 |
| UROS | c.217T>C | 1/75 | 1/83 | 1/159 | 1/47 |
| CEP290 | c.7220_7223del | 1/90 | 1/89 | 1/179 | 1/50 |

CI have been calculated on proportions using the online tool https://sample-size.net/confidence-interval-proportion/ ; * CR calculated using WGS in the present study ; ** One sided 97.5% CI.

**Table C: Case reports on Quebec founder variants.**

| ClinVar ID | Position GRCh38 | CR in SLSJ in this study | Reference |
|---|---|---|---|
| 208402 | chr20:49524089:C:T | 1/56 | Ambalavanan, 2016(5) |
| 197861 | chr4:78252452:C:T | 1/138 | Boissel, 2018(6) |
| 50932 | chr1:152307547:G:A | 1/37 | Brown, 2011(7) |
| 2587 | chr6:18122402:G:C | 1/144 | Chan, 2003(8) |
| 162627 | chr3:20183961:T:C | 1/51 | Chetaille, 2014(9) |

| | | | |
|---|---|---|---|
| 13165 | chr6:42721781:A:G | 1/156 | Coussa, 2015(10) |
| 505170 | chr11:17634152:C:CA | 1/48 | Cruz Marino, 2022(11) |
| 228285 | chr12:80238872:CTA:C | 1/76 | Cruz Marino, 2022(11) |
| 570379 | chr7:143332769:T:C | 1/100 | Dupré, 2009(12) |
| 44121 | chr10:101010781:CT:C | 1/171 | Ebermann, 2010(13) |
| 2326 | chr6:152321898:T:C | 1/276 | Haj Salem, 2021(14) |
| 5360 | chr1:179561327:C:T | 1/179 | Kitzler, 2018(15) |
| 1262 | chr13:50945445:G:A | 1/199 | La Piana, 2014(16) |
| 1273 | chr4:3493106:A:AGCCT | 1/21 | Müller, 2007 ; Srour, 2010(17,18) |
| 217607 | chr4:15599699:A:T | 1/33 | Srour, 2012(19) |

### Table D: Comparison of imputations of ClinVar rare variants using the TOPMed r2 or the Quebec reference panel.

| | TOPMed reference panel | | Quebec reference panel | |
|---|---|---|---|---|
| | **Number** | **Proportion** | **Number** | **Proportion** |
| | **SNPs (total : 1,302)** | | | |
| **Missing in imputed data** | 662 | 0.508 | 18 | 0.014 |
| **Agree in all samples** | 383 | 0.598 | 1070 | 0.833 |
| **Homozygote switch*** | 1 | 0.002 | 3** | 0.002 |
| **False positive*** | 17 | 0.027 | 87** | 0.068 |
| **False negative*** | 212 | 0.331 | 105 | 0.082 |
| **Both false positive and negative** | 27 | 0.042 | 19** | 0.015 |
| | **Genotypes (total : 1,302 SNPs x 1,852 individuals)** | | | |
| **Agree among common SNPs**** | 1184762 | 0.99956 | 2377119 | 0.99964 |
| **Homozygote switch** | 1 | 8.4E-07 | 40 | 1.7E-05 |
| **False positive** | 77 | 6.5E-05 | 142 | 6.0E-05 |
| **False negative** | 411 | 3.5E-04 | 163 | 6.9E-05 |

Proportions are based on common SNPs/genotypes except for missing SNPs which is on the total number of SNPs. False positives are defined as heterozygotes in imputed, but not in WGS data. False negatives are defined as heterozygotes in WGS, but not in imputed data. * In at least one individual. **SNPs having at least one homozygote switch genotype in addition to SNPs having more than 10% of false positive genotypes (101 out of the 106 false positive SNPs) were considered as unreliable in the imputed data.

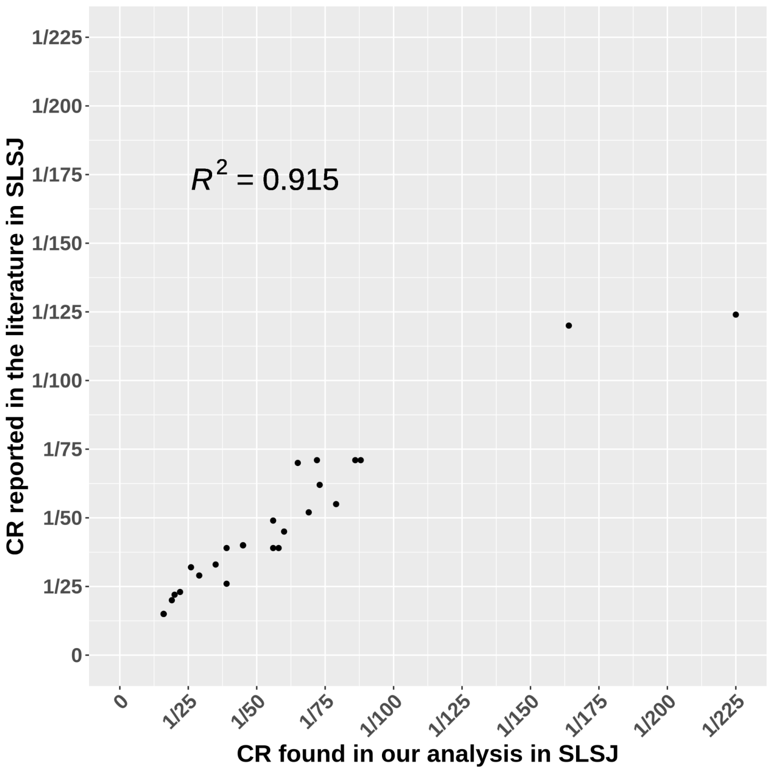


**Figure A: Comparison of the variants' carrier rates reported in SLSJ and found in our analysis.** When available, the aggregated CR (all variants associated with the same disease) was used; also, if available, the CR from the imputed data was used; otherwise, the CR from WGS data was utilized. Variants from the same disease were grouped as in previous studies.

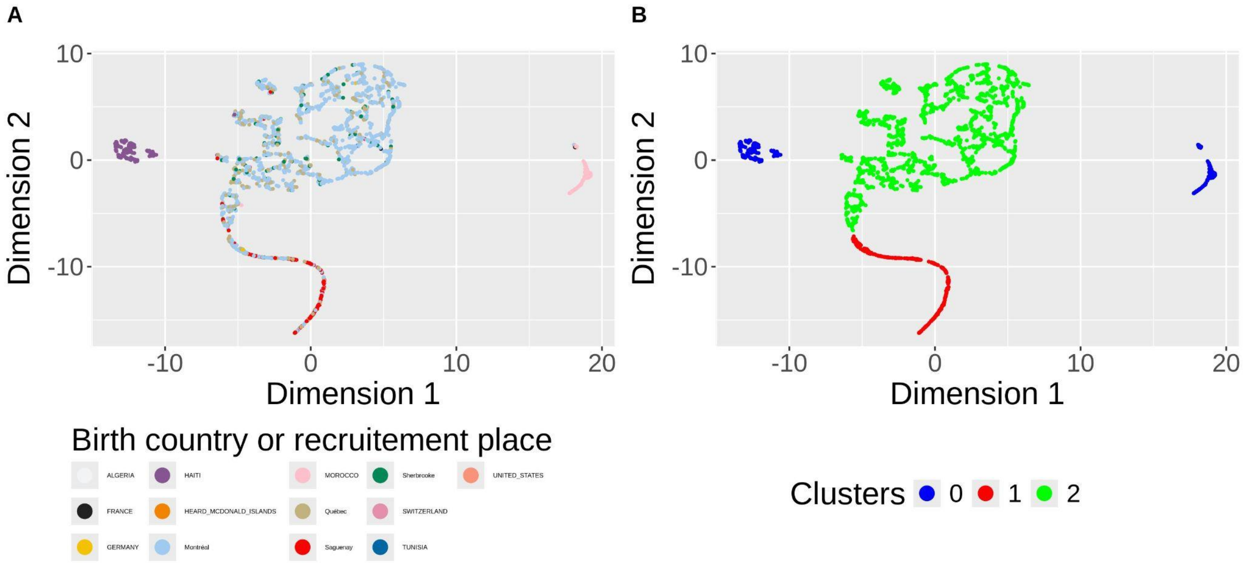


**Figure B: UMAP of WGS data.**
UMAP are coloured according to A) the recruitment region or country of birth and B) the k-means clustering.

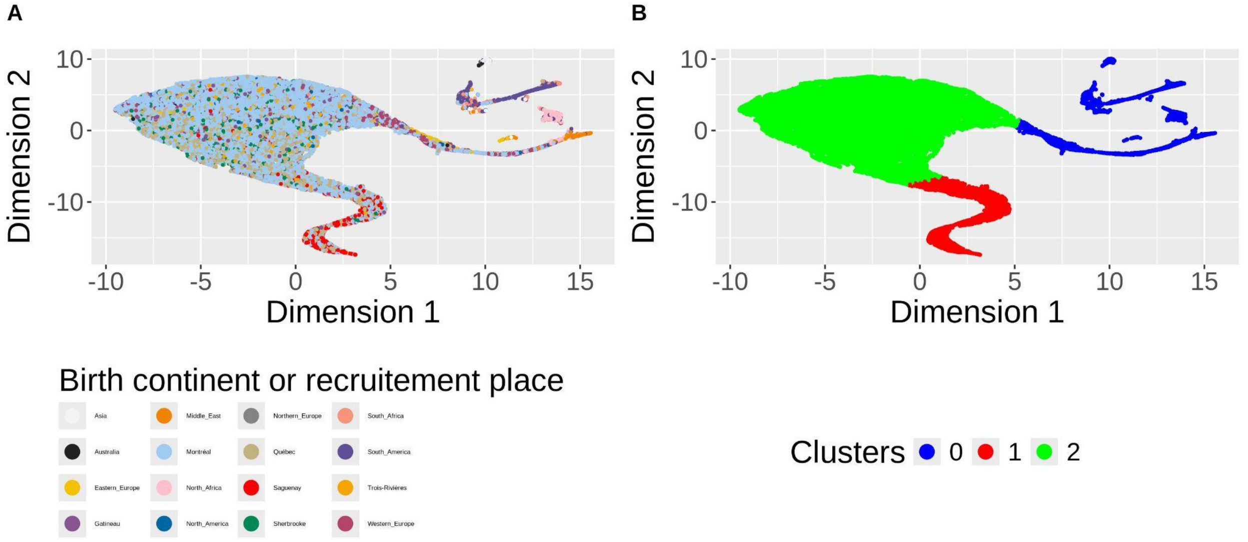


**Figure C: UMAP of imputed data.**
UMAP are coloured according to A) the recruitment region or continent of birth and B) the k-means clustering.

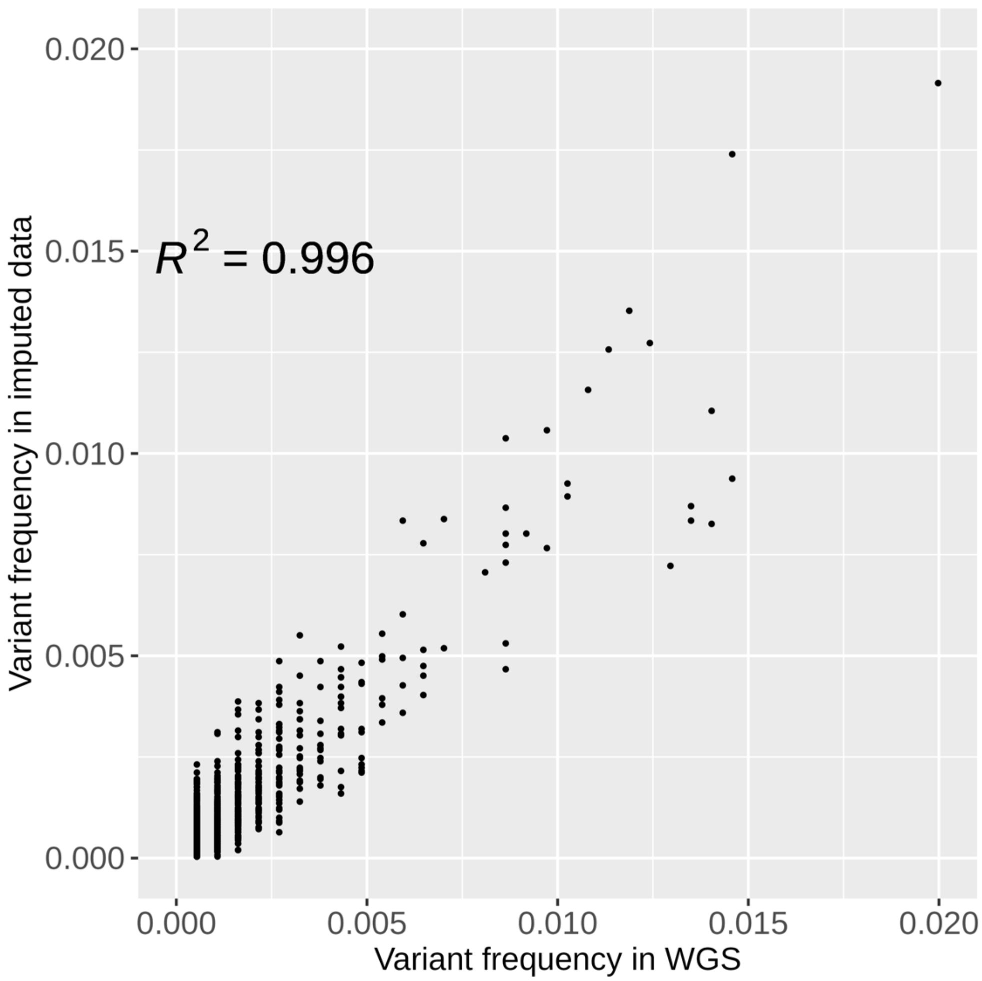


**Figure D: Correlation between imputed and WGS variants' frequency in QcP.**

**Supplementary Methods**

**Experimental validation of carrier rates (Table B)**

Nine variants were selected based on their pathogenicity and expected high frequency in the SLSJ region to validate the carrier rate estimates derived from the imputed data. We randomly selected 1,000 samples from an independent cohort of individuals the SLSJ who had consented to the storage of their anonymized DNA samples for research purposes. They were genotyped using custom TaqMan genotyping assays (catalog #4332072; Applied Biosystem Inc). We designed the assays with specific probes targeting each allele using the Primer Express software. We extracted the DNA from buccal swab samples using DNA extract all kit (catalog #4402616; Applied Biosystem Inc) following the manufacturer recommendations. In brief, 22 µl of Lysis solution was added to 7 µl of buccal swab emulsions, then incubated for 3 minutes at 95°C in a thermocycler. 22 µl of DNA stabilizing solution was then added to the mix. For amplification and detection, the manufacturer recommendations were followed. In brief, for each reaction 1.75 µl of sterile water, 3.10 µl of GTXpress Master Mix (catalog #4401892; Applied Biosystem Inc), 0.15 µl of TaqMan assay, and 1.2 µl of DNA. Analysis was carried out in a 96-well plate and samples were amplified on a 7500 Fast Real-Time PCR thermocycler (Applied Biosystems Inc). The amplification conditions were as follows: 1: 60°C for 1 min with fluorescence acquisition, 2: 95°C for 20 s, 3: 95°C for 3 s, and 60°C for 30 s with fluorescence acquisition (step three was repeated 40 times), 4: 60°C for 1 min with fluorescence acquisition. The genotypes were called using 7500 Software v2.0.1 (Applied Biosystem Inc) after visual inspection of the amplification data.

## References


1. Laberge AM, Michaud J, Richter A, Lemyre E, Lambert M, Brais B, et al. Population history and its impact on medical genetics in Quebec. Clin Genet. 2005;58(4):287–301.
2. Scriver CR. Human Genetics : Lessons from Quebec Populations. Annu Rev Genomics Hum Genet. 2001;2:69–101.
3. Bchetnia M, Bouchard L, Mathieu J, Campeau PM, Morin C, Brisson D, et al. Genetic burden linked to founder effects in Saguenay-Lac-Saint-Jean illustrates the importance of genetic screening test availability. Vol. 58, Journal of Medical Genetics. 2021.
4. Cruz Marino T, Leblanc J, Pratte A, Tardif J, Thomas MJ, Fortin CA, et al. Portrait of autosomal recessive diseases in the French-Canadian founder population of Saguenay-Lac-Saint-Jean. Am J Med Genet A [Internet]. 2023 May 1 [cited 2024 Sep 29];191(5):1145–63. Available from: https://pubmed.ncbi.nlm.nih.gov/36786328/
5. Ambalavanan A, Girard SL, Ahn K, Zhou S, Dionne-Laporte A, Spiegelman D, et al. De novo variants in sporadic cases of childhood onset schizophrenia. European Journal of Human Genetics. 2016;24(6):944–8.
6. Boissel S, Fallet-Bianco C, Chitayat D, Kremer V, Nassif C, Rypens F, et al. Genomic study of severe fetal anomalies and discovery of GREB1L mutations in renal agenesis. Genet Med [Internet]. 2018 Jul 1 [cited 2025 Feb 10];20(7):745–53. Available from: https://pubmed.ncbi.nlm.nih.gov/29261186/
7. Brown SJ, Asai Y, Cordell HJ, Campbell LE, Zhao Y, Liao H, et al. Loss-of-function variants in the filaggrin gene are a significant risk factor for peanut allergy. J Allergy Clin Immunol [Internet]. 2011 Mar [cited 2025 Feb 10];127(3):661–7. Available from: https://pubmed.ncbi.nlm.nih.gov/21377035/
8. Chan EM, Young EJ, Ianzano L, Munteanu I, Zhao X, Christopoulos CC, et al. Mutations in NHLRC1 cause progressive myoclonus epilepsy. Nat Genet [Internet]. 2003 Oct 1 [cited 2025 Feb 10];35(2):125–7. Available from: https://pubmed.ncbi.nlm.nih.gov/12958597/
9. Chetaille P, Preuss C, Burkhard S, Côté JM, Houde C, Castilloux J, et al. Mutations in SGOL1 cause a novel cohesinopathy affecting heart and gut rhythm. Nat Genet. 2014;46(11).
10. Coussa RG, Chakarova C, Ajlan R, Taha M, Kavalec C, Gomolin J, et al. Genotype and Phenotype Studies in Autosomal Dominant Retinitis Pigmentosa (adRP) of the French Canadian Founder Population. Invest Ophthalmol Vis Sci [Internet]. 2015 Dec 1 [cited 2025 Feb 10];56(13):8297–305. Available from: https://pubmed.ncbi.nlm.nih.gov/26720483/
11. Cruz Marino T, Tardif J, Leblanc J, Lavoie J, Morin P, Harvey M, et al. First glance at the molecular etiology of hearing loss in French-Canadian families from Saguenay-Lac-Saint-Jean's founder population. Hum Genet [Internet]. 2022 Apr 1 [cited 2025 Feb 10];141(3–4):607–22. Available from: https://pubmed.ncbi.nlm.nih.gov/34387732/
12. Dupré N, Chrestian N, Bouchard JP, Rossignol E, Brunet D, Sternberg D, et al. Clinical, electrophysiologic, and genetic study of non-dystrophic myotonia in French-Canadians. Neuromuscul Disord [Internet]. 2009 May [cited 2025 Feb 10];19(5):330–4. Available from: https://pubmed.ncbi.nlm.nih.gov/18337100/
13. Ebermann I, Phillips JB, Liebau MC, Koenekoop RK, Schermer B, Lopez I, et al. PDZD7 is a modifier of retinal disease and a contributor to digenic Usher syndrome. J Clin Invest [Internet]. 2010 Jun 1 [cited 2025 Feb 10];120(6):1812–23. Available from: https://pubmed.ncbi.nlm.nih.gov/20440071/
14. Salem IH, Beaudin M, Stumpf M, Estiar MA, Côté PO, Brunet F, et al. Genetic and Epidemiological Study of Adult Ataxia and Spastic Paraplegia in Eastern Quebec. Can J Neurol Sci [Internet]. 2021 Sep 1 [cited 2025 Feb 10];48(5):655–65. Available from: https://pubmed.ncbi.nlm.nih.gov/33397523/
15. Kitzler TM, Kachurina N, Bitzan MM, Torban E, Goodyer PR. Use of genomic and functional analysis to characterize patients with steroid-resistant nephrotic syndrome. Pediatr Nephrol [Internet]. 2018 Oct 1 [cited 2025 Feb 10];33(10):1741–50. Available from: https://pubmed.ncbi.nlm.nih.gov/29982877/
16. La Piana R, Tran LT, Guerrero K, Brais B, Levesque S, Sébire G, et al. Spastic paraparesis and marked improvement of leukoencephalopathy in Aicardi-Goutières syndrome. Neuropediatrics [Internet]. 2014 Dec 1 [cited 2025 Feb 10];45(6):406–10. Available from: https://pubmed.ncbi.nlm.nih.gov/25343331/
17. Müller JS, Herczegfalvi A, Vilchez JJ, Colomer J, Bachinski LL, Mihaylova V, et al. Phenotypical spectrum of DOK7 mutations in congenital myasthenic syndromes. Brain [Internet]. 2007 Jun [cited 2025 Feb 10];130(Pt 6):1497–506. Available from: https://pubmed.ncbi.nlm.nih.gov/17439981/
18. Srour M, Bolduc V, Guergueltcheva V, Lochmüller H, Gendron D, Shevell MI, et al. DOK7 mutations presenting as a proximal myopathy in French Canadians. Neuromuscul Disord [Internet]. 2010 [cited 2025 Feb 10];20(7):453–7. Available from: https://pubmed.ncbi.nlm.nih.gov/20610155/

19. Srour M, Schwartzentruber J, Hamdan FF, Ospina LH, Patry L, Labuda D, et al. Mutations in C5ORF42 cause Joubert syndrome in the French Canadian population. Am J Hum Genet. 2012;90(4).
