# Supplementary material for: Rare diseases load through the study of a regional population
Source: PLoS Genet. 2025 Oct 16;21(10):e1011876. doi: 10.1371/journal.pgen.1011876 (PMC12530595; doi:10.1371/journal.pgen.1011876)
Supplement: Attachment — Submitted filename: rebuttal_letter.pdf [file pgen.1011876.s004.pdf]

We first want to thank the reviewers for their insightful comments on our manuscript. We are happy to report that we were able to address all of the comments and we provide here a point-by-point review to each specific point that was raised.

Reviewer #1

Major comments

1. The analysis about clinical validation is very interesting, and perhaps the highlight of the paper, but it should be expanded. First, how large were the external databases, and is there any chance that patients found by clinicians were also used in the analysis ("train-test contamination")? How many variants were sent for validation and how many of those ~30 people were previously undiagnosed? If any, that needs highlighting. Some of these questions might have obvious answers (or no answer due to privacy constraints), but more details should be given to help assess the diagnostic utility of the findings.

As all CaG samples are anonymized, determining any potential overlap between them and the patients is not possible. However, we don't expect an overlap since these are two separate cohorts.

This section has been clarified in the text. The goal was not to establish a diagnosis, but rather to identify patients carrying previously undocumented founder variants in the SLSJ, and who had already been diagnosed with the corresponding disease. This served as a proof of concept, demonstrating that our population-based approach can aid in uncovering diseases of potential relevance to community health without waiting for a sufficient number of patients to show up in the clinic.

Also see answer to reviewer #3, major point #1.

2. The way the authors compare their list of founder variants with those previously reported is confusing. They first describe 42/72 reported previously (and found here), then mention "38 of the (80) founder variants were already documented…". How do the 38 variants compare with the "42 previously reported" given in Table 1? Please consider restructuring this section for clarity by first presenting your findings, then indicating which are novel and which overlap with prior studies.

We recognize that this section was confusing and have revised the text for clarity. We also removed Table 1 from the manuscript, as it primarily listed previously reported variants that were already included in the Supplementary Table 1. This allowed us to focus the results section on the novel variants that were originally presented in Table 2, which is now renumbered as Table 1. The number 42 coincidentally appears twice, but it refers to completely different sets of variants. The first 42 variants (old Table 1) refer to those previously reported in four literature reviews focused on the SLSJ founder effect[1–4]. In contrast, renumbered Table 1 reports 42 founder variants that were never mentioned in the literature reviews **or in any case reports**. We conducted a literature search specifically for case reports involving only variants classified as founders according to our criteria. We did not search case reports for the whole set of variants with RFD>=10%.

3. A common choice for comparing two groups is a two-samples t-test. Instead, the authors report p-values from chi2 tests. This choice should be justified, and the statistical tests described in more detail in Methods.

In this case, we compared two proportions rather than two means. Specifically, the proportion of individuals who do not carry any variants (or who carry two or more variants)

relative to the total number of variants in each of the 2 groups. Since these proportions are categorical variables, the chi^2 test is an appropriate method for their comparison in a 2X2 contingency matrix. In contrast, a t-test is typically used to assess differences between means of continuous variables.

4. Using IBD sharing to clean founder variants is an interesting feature, but how did that help on top of the CR filter? Is there a reference paper that did something similar? The authors should justify the use of 50% as threshold, and how sensitive their analysis is.
We agree with Reviewer #1 and 3 that IBD analysis is a valuable tool for identifying founder variants, as it helps determine whether a variant likely originates from a single, or a small number of recent common ancestors, as described in our Methods section lines 385-389.
Currently, there is no universally accepted definition of a founder variant in the literature. Most studies rely primarily on the frequency of a variant within the ‘source’ and ‘founder’ populations, as seen in the four reviews cited in our manuscript[1–4] and in more recent publications[5–7].
To our knowledge, no previous studies have specifically used the amount of IBD sharing around causal variants for this purpose, although several have investigated founder haplotypes[8–12] or ancestral recombination graphs[6] which are similar.
We figured out the 50% threshold using the data. Among the previously reported variants listed in the four reviews and having a CR above 1/200 in the SLSJ, we observed only 1 below our 50% threshold (chr7:117559590:ATCT:A). However, this variant is also relatively common in the non-Finnish European population (allele frequency of 0.015 in gnomAD v4.1.0), which also often serves as an additional criterion for ruling out a variant as a founder variant in other studies and supports the multiple introductions by many different founders. See figure 1 below.
Also see answer to reviewer #3, major point #3.

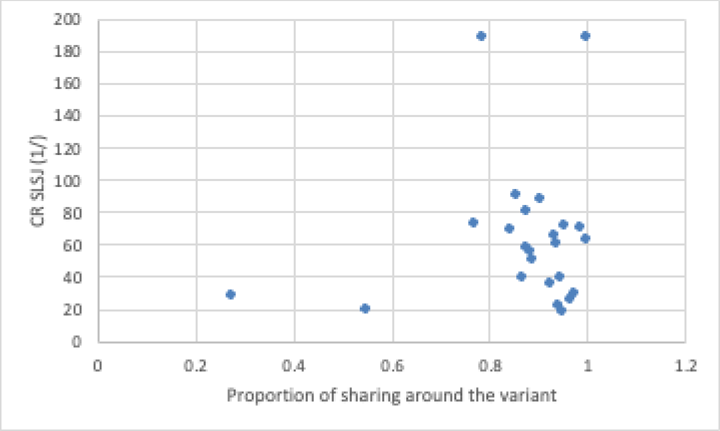

Figure 1: Proportion of IBD sharing compared to CR for variants reported in reviews having a CR above 1/200 in SLSJ

Minor comments
• The section “Experimental validation of carrier rates” is misleading, as experimental usually refers to lab work carried out to validate one of the main findings. Effectively what the authors did was targeted genotyping to verify that their statistical imputation was accurate. Nice to have, but I believe that is more of a supplementary analysis.
We put this in the supplementary as suggested.
• Please convert Supplementary Table 1 to csv rather than PDF.

This was done.
• L277: “most related individuals” is that a typo? PCA needs to be performed in a set of unrelated individuals, and then any relatives need to be projected on the principal components.
This was rephrased.
• Table captions should be expanded to include definitions (e.g., F/NF) and sample sizes, particularly for Table 1.
This was done.
• There are references mentioned twice, e.g. 10 & 21 or 13 & 18, perhaps more.
This was corrected, thanks for noticing.
• Consider citing Ishiki et al 2024 (PMID: 39399040) who performed a similar study.
This was added, line 68.
• L319: should be 1,302 variants.
We found 1,304, but removed 2 due to MAF>0.05 as stated in the next sentence.
• P-values are not written in the correct format, or contain typos.
This was corrected.

Reviewer #2

Major Points:

1. Lines 99-101. Given that many more WGS and imputed samples were available for the UQc than SLSJ cluster, (314 WGS and 3,589 imputed from SLSJ, or 1,538 WGS and 21,472 imputed from UQc), it is unsurprising that many more of the RFD >= 10% variants would be unique to UQc vs SLSJ. Regarding this, the authors state in the Discussion that “Indeed, 42% of variants with an RFD≥10% in the QcP were lost in the SLSJ, although some of them might be too rare to be observed in the SLSJ due to the smaller sample size” (lines 200-201). To better compare the count or proportion of variants missing from SLSJ and UQc, the authors should as a separate analysis down-sample the UQc cohort to match the SLSJ sample size, or repeatedly bootstrap equal-size subsets.

We thank the reviewer for their suggestion. We resampled UQc down to 3,589 individuals 1,000 times and calculated the average number of variants lost in UQc at this sample size. This was also done for Figures 2 and 5 in the manuscript. Moreover, for these analyses we retained only variants with imputed data, as the two data types were based on different sample sizes that might include some bias.

2. Lines 99-101. Additionally, the cited missing variant counts (540 from SLSJ and 17 from UQc) are not shown in Figure 1, or anywhere else that I can find. It would be useful to provide a table summarising the counts of potentially pathogenic RFD >= 10% variants coming from SLSJ, UQc, and QcP, and how many are unique to that cluster.

All 1,302 variants with an RFD ≥10% are comprehensively listed in supplementary Table 1, which includes their group-specific CR, founder status, and additional details. For easier access and review, this table will be provided in another format. This analysis was also repeated using resampling for UQc to account for the different sample sizes.

3. Lines 99-101. It is possible to visually confirm from Figure 1 that many variants are much more frequent in SLSJ than gnomAD NFE, seen on the right half of Figure 1b close to the X axis, whereas this is not the case for the combined QcP cluster in Figure 1a. I am wondering, however, if there is a better way to quantify that “many variants are more frequent in the SLSJ region”, such as a table which counts the number or proportion of the 1,302 variants above a certain RFD or fold-increase threshold for each cluster? How many variants, for instance, have a >=50% or 90% RFD vs gnomAD NFE in the SLSJ or QcP clusters?

The number of variants distributed across different RFD thresholds is consistently lower in SLSJ, primarily because UQc harbors a greater number of variants overall due to its greater sample size (see Table 1 below). The vast majority of variants have an RFD greater than 0.8 for both groups. Additionally, RFD does not necessarily correlate with variant frequency as shown in Figure 2 below. The difference in the mutation load between SLSJ and UQc (or QcP) lies not in the number of variants with different RFD, but rather in the elevated frequency or CR of a minority of them in the SLSJ which is what we wanted to represent in Figure 1 of the manuscript.

Table 1: Number of variants in each RFD bins

RFD	No variants in SLSJ	No variants in UQc
0.1-0.2	8	10

0.2-0.3	15	23
0.3-0.4	5	8
0.4-0.5	34	45
0.5-0.6	17	19
0.6-0.7	49	70
0.7-0.8	25	36
0.8-0.9	196	375
0.9-1	412	699

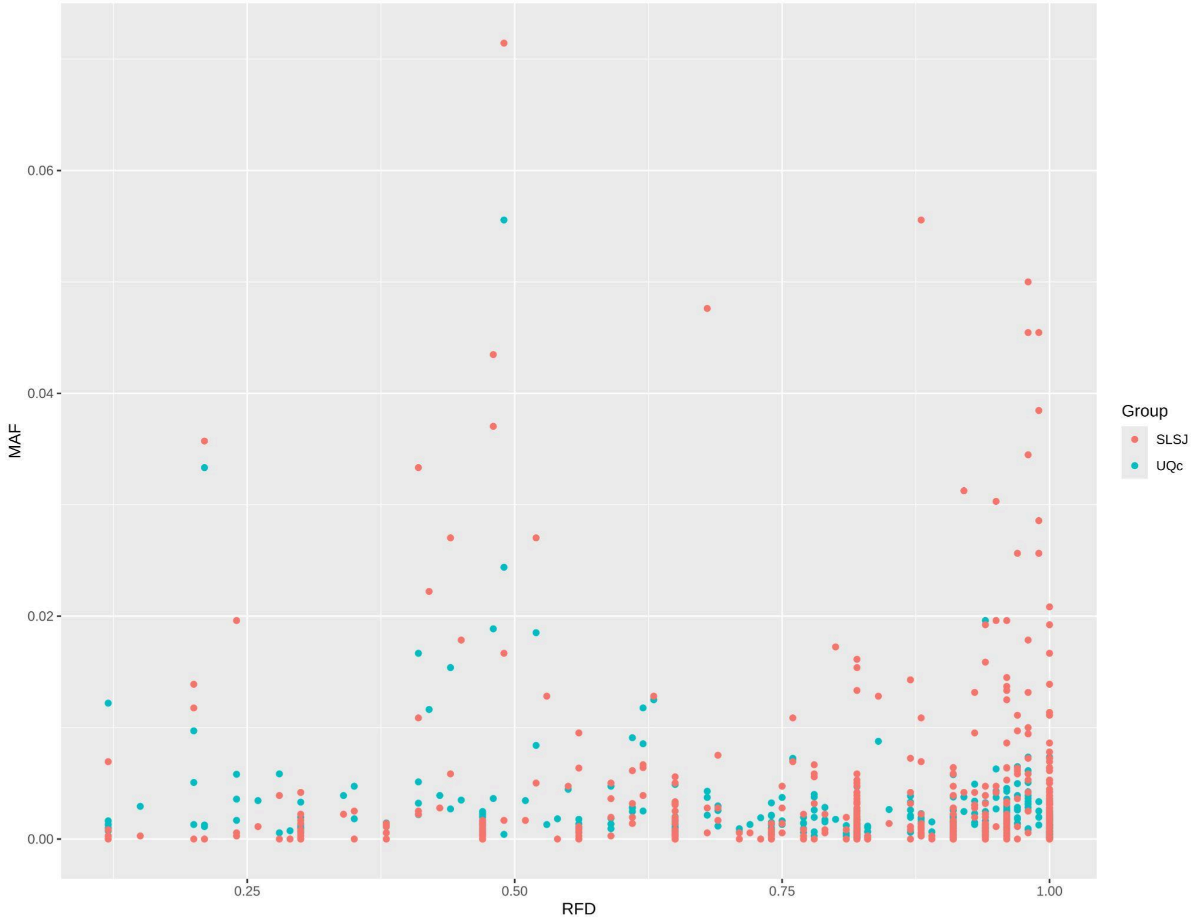

Figure 2: MAF and RFD (>= 10%) across both groups

4. Lines 101-103. Figure 2 plots the count and proportion of individuals from the SLSJ and UQc (rest of Quebec) clusters which carry 0, 1, 2, 3 … 9 of the 1,302 rare potentially pathogenic >= 10% RFD variants. Given that this figure primarily seems to shed light on the mutational load in SLSJ vs UQc, I am wondering whether the >= 10% RFD threshold is necessary? Why not repeat the same analysis for all rare, potentially pathogenic variants (as defined by ClinVar and previous SLSJ publications), regardless of RFD threshold? Additionally, I find it striking that, for SLSJ, only ~13% of individuals carry none of the 1,302 rare, potentially pathogenic >= 10% RFD variants of interest (and ~27% of UQc individuals). Because many of these variants are likely to come from ClinVar P/LP entries with just 1*

review status, it would be informative to repeat this analysis with only ClinVar P/LP variants with >= 2* review status (plus the previously reported founder variants).

The 10% RFD threshold was used as an initial filtering step to narrow down the ~200,000 ClinVar pathogenic variants. The 10% cutoff was chosen to ensure inclusion of all known variants in the four reviews[1–4] , with the lowest RFD among these being 0.21 for the chr7:117559590:ATCT:A variant. There were 442 variants with RFD < 10% in the WGS and most of them were below 0.005 frequency both in SLSJ and UQc (See Figure 2 below). It can be noted in supplementary Figure 2 of the manuscript that most of the variants with RFD < 10% (in WGS exclusively) are very rare in the QcP. They would not influence the load imbalance that we observed between the SLSJ and UQc with the RFD >= 10%. Moreover, many of these were not present in the imputed data and would probably be too rare to be of interest in SLSJ given its smaller sample size. We then decided not to include them in the analysis since the goal was not to perform a systematic analysis of all pathogenic variants.

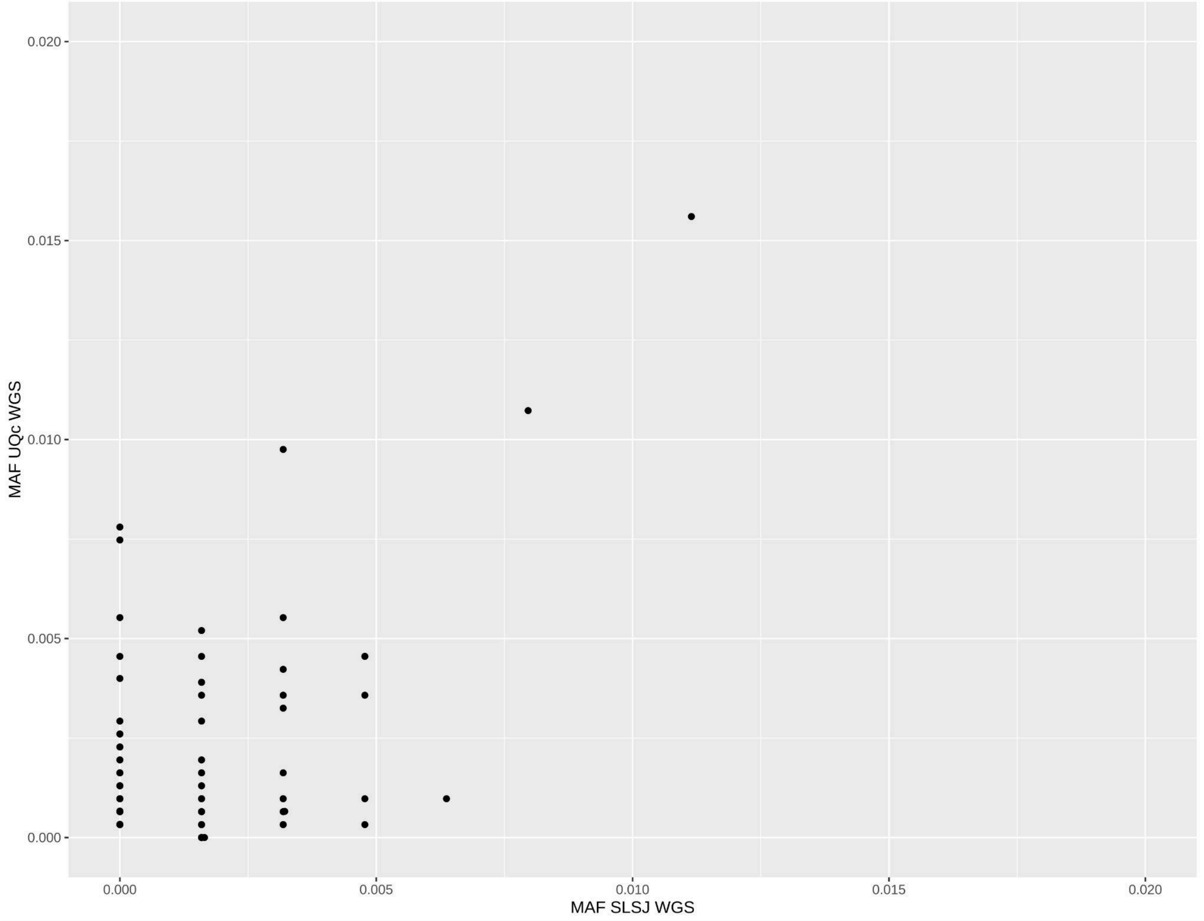

Figure 3: MAF of variants with RFD < 10% in WGS

The reviewer is correct that ClinVar variants with a review status of ≤1* may not be clinically relevant yet. However, we chose not to filter based on this criterion because 11 previously documented variants fall into this category. Excluding them could have unnecessarily limited our results, especially given that ClinVar is continuously updated and these variants are very rare elsewhere in the world. Moreover, we identified 12 new founder variants with this review status, some of which being very frequent in the SLSJ population. Notably, one variant (EIF2AK4 c.1153dup) was observed in a homozygous state in a patient presenting with the associated phenotype. The aim of our study was not to diagnose individuals, but to identify

variants present in the population that may be linked to a founder disease. Clinical validation will be performed in collaboration with healthcare professionals to determine the true clinical significance of these variants in further stages of this project.

5. Lines 113-115. I cannot find any mention elsewhere in the manuscript or table/figure legends as to how the nine variants were selected to have their carrier rates reassessed, and whether this is an adequate number of variants to use for validation. Additionally, the authors should clarify (either in lines 113-115 or lines 359-361) whether the "subset of 1,000 randomly selected samples with appropriate consent" are a subset of the CARTaGENE individuals from SLSJ used in the SLSJ WGS/imputed datasets, or other individuals from SLSJ not used previously in the study. The term "subset" from lines 113-115 quoted above suggests the former, but the title of Supplementary Table 3 "Experimental reassessment of CR in an independent cohort of 1,000 individuals living in SLSJ" suggests the latter. If the 1,000 SLSJ subset does in fact come from the CARTaGENE 314 WGS and 3,589 imputed genotypes from SLSJ, then the authors should report the precise % of matching genotypes between the validation subset and the original data.
Following Reviewer #1's suggestion, this section was moved to the supplementary material. The 1,000 individuals from the SLSJ cohort used in this analysis were independent of the CARTaGENE dataset; this has been clarified in the revised supplementary text. Since CARTaGENE and the independent SLSJ cohort are anonymized, determining any potential overlap between them is not possible, but we do not expect any overlap.
In fact, we leveraged this data from another lab's project in the CIUSSS of the SLSJ to provide supplementary validation for some of our CR. The 9 variants were selected by the CIUSSS lab based on their pathogenicity and their anticipated high frequency in both the current study and previous research. This was also added in the supplementary text. However, we acknowledge that including more variants in this validation would have strengthened the analysis.

Minor Points:
6. Lines 59-66. The first paragraph of the Introduction gives some background on the prevalence of rare diseases (thought to affect 10% of population, more than 10,000 rare diseases in Orphanet). It would be useful to specify that the authors are mainly concerned with rare Mendelian diseases.
This was added, lines 25 and 60.
7. Lines 133-135. The authors should comment on whether any variants were more common in UQc than in SLSJ, and how many.
Only 16 variants were at a higher CR in UQc than in the SLSJ among the 1,302 variants with RFD≥10%. This was added in the text, line 138.
8. Lines 136-139. Because inheritance patterns are reported for many of these 80 founder variants (some are shown in Table 2), it would be useful to estimate the expected count or rate of affected individuals, in addition to the observed carrier counts shown in Figure 5.
For instance, a variant with a carrier rate of 1/200 for a recessive disease in a population of 286,768 (the SLSJ population in 2024) would be expected to result in approximately 7 homozygous affected individuals; this was added in the method section, lines 368-369.
Since the focus of our study was not on patients but rather on using population data to identify potentially high-frequency pathogenic variants within the population and given the already dense content of the tables, we chose not to include this information in the tables.

9. Figure 4. The X axis labels are unusual, each ending in an open square bracket where it should be a closed square bracket. Additionally, I am confused why many UQc founder variants are shown with carrier rates below 1/200, given that a carrier rate above 1/200 was a requirement for founder variants. The authors should specify whether the label “UQc founder variants” here means variants which are founder variants in UQc, or something else, like founder variants in any region with their UQc carrier rate.
The figure was corrected, legend changed and details added in the caption for more clarity. The reviewer is correct that the founder variants with CR below 1/200 are founder in the other group only.
10. Lines 154-156. The nucleotide change and phenotype are listed, but authors should also list the associated gene name.
This was added.
11. Table 3. The table is described as having corresponding phenotypes, but no actual phenotypes are listed.
This was added and this table was updated with the most up-to-date results from the clinic.
12. Table 3. Nine instances of compound heterozygotes in patients are documented, also referred to in lines 171-174 of the Discussion. It would be useful to note the other variant in each case, and also to comment on whether it was possible to determine whether the two observed variants were in trans (a true compound heterozygote) or in cis on the same haplotype.
This information was added to the table. All compound heterozygous variants were located within the same gene and in close proximity to the variant of interest (in cis), but their different frequencies make it difficult to determine whether they are on the same haplotype. For example, both variants in the PDZD7 gene were identified with CR of 1/171 (c.2107del) and 1/26 (c.2672AGA[1]). Additionally, the second variant in the UROS gene, c.424C>T, was not detected in this study, suggesting it may be very rare.
13. Lines 272-273. The final imputed dataset appears to have more individuals (29,353) than the number of initial CARTaGENE chip genotypes (29,337, mentioned lines 252-253). I am wondering if this refers to the number of imputed genotypes + the WGS, after filtering for sample missingness—the language is ambiguous. However, the subsequent application of the imputed dataset to refine the Quebec allele frequencies suggests the imputed and WGS datasets were not merged, as the authors mention that imputed frequencies only superseded WGS frequencies when the variant was present in the imputed dataset.
This was a mistake and was corrected, there were 29,353 (not 29,337) genotyped samples available that were imputed, thanks for noticing. The WGS samples were also imputed from the genotypes, so they were all included in the imputed dataset. This was added in the text, line 316.
14. Lines 299-302. I presume that “conflicting (both pathogenic and likely pathogenic variants)” simply refers to variants with an aggregate germline classification of “Pathogenic/Likely pathogenic”, and not to “Conflicting classifications of pathogenicity”, as the latter term implies variants with both pathogenic and uncertain/benign submissions. If so, I would advise removing the word “conflicting” from the authors’ description.
This was corrected, the reviewer is right, it is not conflicting pathogenicity, but both pathogenic/likely pathogenic.
15. Methods for calculating RFD, lines 306-321. As far as I can tell, closely related individuals were not removed from the SLSJ, UQc and QcP cohort clusters used to calculate various Quebec variant frequencies. If this was done, it should be stated, and if not, the choice should be explained.

Individuals from CARTaGENE were randomly recruited in the population, so the close relatives were not excluded from our CR calculation. We found 0.003% of the SLSJ pairs who shared more than 1500cM total IBD and 0.0003% for UQc.
To ensure that the reported carrier rates were not inflated due to multiple closely related individuals carrying the same founder variant, we recalculated all carrier rates by randomly removing one individual from each pair sharing a total length of more than 1500cM. We found that this only marginally changed the carrier rates. We opted to keep the original carriers to preserve the complete structure of our population since randomly removing individuals could have more impact on other variants. Four variants were found to be slightly under the 1/200 threshold (clinvar# 2980542 was founder in the SLSJ ; clinvar# 3117894 was founder in UQc and clinvar# 2298 and 68815 which were already NF in UQc). Two of them were already not founder due to other criteria and the two others were not discussed in the article.

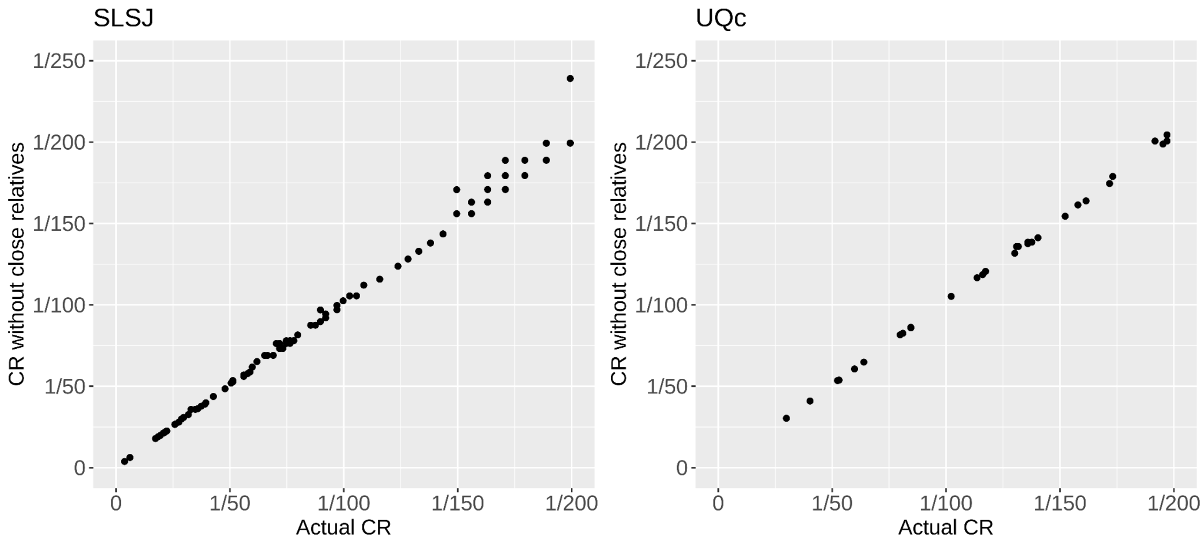

Figure 4: CR of founder variants without close relatives

16. Methods for calculating RFD, lines 306-321. It would be useful to comment on the statistical significance of an observed RFD of 0.1 between any of the SLSJ, UQc, and QcP cohorts and the gnomAD v.3.1.2 NFE genomes, for a variant at some of the gnomAD or Quebec/SLSJ allele frequencies relevant to this study. Additionally, for ease of reading, the authors should briefly state in the Methods or Results what an RFD of 0.1 would translate to in terms of fold-difference in frequency between the SLSJ/Quebec population and gnomAD NFE, perhaps for other reference RFDs as well, such as RFD = 0.5 or RFD = 0.9. For instance, RFD = 0.1 corresponds to a 1.11x greater frequency and RFD=0.5 corresponds to a 2x greater frequency.
Note that RFD was only calculated on the Quebec province not to have a bias toward the variants at high frequency only in the SLSJ. The fold-difference was added in the method section, line 347.
17. Line 320. It would be useful to name the two variants with MAF >= 0.05, and to state how many of the remaining variants have, for instance, MAF >= 0.01 or MAF >= 0.005.
This was added in the text, lines 352-353. Since MAF is directly related to CR, it is provided for each variant across all cohorts in Supplementary Table 1.
18. Lines 328-331. The authors explain that alternative observed carrier count thresholds of 5, 8, and 10 were used for the SLSJ, UQc, and QcP WGS clusters, due to the smaller

sample sizes. The authors should clarify how exactly thresholds of 5, 8, and 10 were chosen.
This was addressed in the text, lines 365-366. Due to varying sample sizes, the number of individuals corresponding to a CR below 1/200 differed between groups. For the SLSJ population, we chose not to include variants found in fewer than five individuals to minimize the risk of false signals and because IBD sharing patterns were unclear with such low counts.
19. Methods for IBD analysis, lines 336-350. I believe the comparison of pairwise IBD sharing at variant sites was done for each cluster (SLSJ, UQc, and QcP), as founder variants are reported for each in the Results section. Reading this section, particularly lines 343-346, I initially had the impression that the analysis was only done for SLSJ. This should be clarified.
The reviewer is right; this analysis was conducted separately for carriers within the different clusters. This clarification has been added to the text, line 382.
20. Supplementary Table 1. Although this table is not meant to be read in its entirety, it is basically useless in the format it was sent to me for finding out anything about a variant's status in SLSJ, as the variant info (ClinVar ID, Position GRCh38, Data type) for each variant are on different pages (1-25) than the SLSJ columns (26-49). It would also be good to include the gene name or symbol for each variant.
This table will be provided in another format. Genes were also added in the table as suggested.

Reviewer #3

Major comments:

1 • The results have implications for preconception carrier screening in Quebec and particularly in SLSJ. However, the authors don't make specific recommendations for how such a panel should look like, which, unfortunately, somewhat diminishes the impact of the paper. It would be important to describe how such a panel would compare to existing panels in terms of the variants included and the number of couples at risk. (For example, see, https://www.nature.com/articles/gim2015123, although it is a bit dated and using a much smaller dataset).

We have expanded the discussion section, lines 199-212, to include insights on the impact of genetic testing in the region, referencing a recently published study[13] and highlighting how our approach can contribute to these efforts. While identifying pathogenic founder variants at elevated frequencies in specific regions alone does not warrant their immediate inclusion in carrier screening panels, we are actively collaborating with colleagues at the CIUSSS of the SLSJ to explore how our population-based strategy can be integrated into clinical practice. This initiative aims to strengthen the case for approval of additional variants for inclusion in the carrier screening offered to the SLSJ population.

2 • Some of the analyses are difficult to interpret, given that they are based on the same dataset used for discovery. The variants discovered are conditioned, by definition, to have certain frequencies in the discovery dataset. Thus, all results reporting variant counts (Figures 2-5) are somewhat biased (regression to the mean/winner's curse). In other words, variants that made it to the list may have happened to be those "lucky" enough to cross the frequency threshold. However, in another dataset, their frequency will be lower. This is easily solvable by dividing the dataset into discovery and analysis, whereby variants will be discovered in the (larger) discovery subset, and variant counts will be reported based on the (smaller) analysis subset. I would also suggest that the analysis subset will have an identical number of genomes from SLSJ and UQc. Otherwise, again, all variant counts are difficult to interpret.

Variant discovery was conducted using the 1,852 WGS dataset, while the variant counts (except in a few cases) are primarily based on the imputed dataset of 25,061 individuals, To increase our sample size and our precision in the frequency calculations of rare variants. Although we did not remove the individuals having a WGS from the imputed data since they were also imputed from genotypes and were representing a minority of samples. Moreover, the CR observed in this study were consistent with both previously reported values and newly assessed CRs (for nine variants evaluated in an independent cohort at the CIUSSS). Additionally, CRs from the WGS and imputed datasets showed strong correlation.

The goal was not to make a comprehensive list of all variants associated with rare diseases as we believe that our analysis is underpowered to identify diseases with very low carrier rates. This study serves as a proof of concept, demonstrating that our population-based approach can aid in uncovering diseases of potential relevance to community health without waiting for a sufficient number of patients to show up in the clinic, but also to better characterize rare diseases with higher carrier rates amongst a specific population.

The reviewer is right that the variants that were not found in the imputed dataset, but only in the WGS must be considered with caution. We decided to keep these because 3 of the previously reported variants were not found in the imputed dataset (chr17:3655305:G:A, chr12:868772:C:T, chr12:102844356:A:G) and we wished to keep as many known variants

as possible. But for figure 2, 4 and 5, it is true that the mixed imputed and WGS variants can include biases. Thus, as suggested, we removed the variants with WGS data only from these analyses and kept the 1,153 imputed variants with RFD>=10%. Additionally, we resampled 1,000 times individuals in the UQc group to match the sample size of SLSJ for figure 2 and 5. Regarding Figure 4, the point is evident in the first two CR categories (1/14–1/99 and 1/100–1/199), which are significantly more frequent in the SLSJ region despite the smaller number of individuals and variants. Therefore, resampling the UQc cohort would not change this conclusion.

3 • It is difficult to justify the criterion of having a relative frequency difference greater than 10%. Figure 1 shows clearly that many variants are quite common in other Europeans, but have simply drifted to somewhat higher frequencies in Quebec. I would not call these founder variants. The founder variants are those on or close to the x-axis in Figure 1, namely variants with near-zero frequency outside Quebec that have reached a considerable frequency in Quebec. I think that more reasonable criteria would be 5x or 10x fold higher frequency in Quebec, along with frequency in gnomAD under (say) 1/1,000 or 1/10,000. Or the authors could use a method such as this paper: https://journals.plos.org/plosgenetics/article?id=10.1371/journal.pgen.1007329. Perhaps more generally, defining a first set of variants with RFD>0.1 and then a second set that also has a minimal carrier rate and high levels of IBD is quite confusing. I think it is really the second set that is interesting. The IBD analysis is very nice and important in this context.
We agree with the reviewer that the RFD>=10% is not a sufficient criterion to define a founder variant. We apologize for being unclear in our explanation in the manuscript. This threshold was used as an initial filtering step to narrow down the ~200,000 ClinVar pathogenic variants. The 10% cutoff was chosen to ensure inclusion of all previously described founder variants, with the lowest RFD among these being 0.21 for the chr7:117559590:ATCT:A variant. Similarly, the lowest RFD among the variants identified as founder in our study is 0.2 (chr11:64759751:G:A). However, the RFD was calculated across the entire province of Quebec to avoid biasing the analysis toward founder variants specific to the SLSJ region. We therefore found it valuable to first present the broader set of variants that are more frequent across the province, before focusing on our stricter definition of founder variants that are notably more frequent in the SLSJ region compared to urban areas. We renamed the first section of the results, line 93, to make it clearer that we are not talking about founder variants yet.
It is also important to note that there is currently no universally accepted definition of a founder variant in the literature. Commonly, a variant is considered to be associated with a founder effect when it is observed at elevated frequency within a genetically related group and can be traced to one or few recent common ancestors[5–7]. In our study, in addition to the frequency criterion, we assessed IBD sharing at the variant locus among carriers as noted by the reviewer. A high proportion of pairs of carriers sharing the same IBD segment at the variant's location indicates that it was likely inherited from a recent common ancestor and subsequently increased in frequency, consistent with a founder effect. Our definition of founder variants is therefore more stringent than others and results in the identification of fewer variants. In fact, some historically recognized founder variants in the SLSJ were not classified as such in our study. This was added in the discussion, lines 183-190.
Also see answer to reviewer #1, major point #4.

4 • The clinical data analysis is missing many details. What was the sample size? Was there ethical approval for the search? Who did the search? Which variants were considered? In addition, Table 3 seems to contradict the associated text, as all the three variants mentioned in the text were seen only once based on Table 3, while line 159 suggests a variant is of medical interest only if seen three times. In Table 3, which variants are already in screening panels? More broadly, it is not clear what is the motivation for this analysis and what we actually learn from it. These variants are known to be pathogenic and they were found in the discovery dataset. So what new information do we learn from this review of medical records?
All this information has been added to the text and the whole results section, lines 159-173, was revised for clarification. All of the variants are classified as pathogenic, however, some still lack strong supporting evidence in ClinVar (1-star ratings), despite being included in diagnostic panels. Our population-based study may help highlight variants that warrant further investigation.
The reviewer is right in pointing out a contradiction, this was our mistake. In fact, there is no formal criterion requiring three unrelated patients for a variant to be considered for inclusion in a carrier test. Instead, it is the data generated in the present study that provided the CIUSSS Medical Genetics Service with additional evidence of a higher frequency in the region, helping to inform and refine the new carrier test design. This reflects the objective of our work: to collaborate with the clinic in building a more comprehensive picture of regionally founder variants, ultimately enhancing carrier testing for the population. The clinical databases review is also a validation that our population-based approach is useful in this context since it identifies diseases that are present in the region and observed in the clinic. Also see answer to reviewer #1, major point #1.

Minor comments:
• Lines 99-100: this result is likely due to the imbalance in sample size between SLSJ and UQc.
This has been corrected, see answer to reviewer #2, major point #1.
• Please add the y=x line to Figures 1 and 3.
This was done
• Table 1. What is the “status” column? Please also add the genomic coordinates of the variants (at least in a supplementary table, if space in the main text is limited).
Table 1 was removed from the manuscript, all information being now in supplementary Table 1. The genomic coordinates are also provided in the supplementary table 1 and gene names have been added to facilitate searches. This table will also be provided in a more convenient format.
• It will be interesting to see the distribution of IBD fraction across the 1302 variants of the first set and the 80 variants of the second set. This will help understand whether and how this filter is helping to identify the founder variants.
To improve clarity in the article, we chose to present this information in the supplementary table 1, as each variant requires a separate graph illustrating the proportion of carriers’ pairs sharing IBD at each genomic position. Some graphs are provided in figure 5 below. The red line indicates the position of the variant. For almost all the variants shown, there is a peak of IBD sharing around the variant position that exceeds 0.5, the threshold we used to indicate that at least half of the carriers’ pairs share around the variant (see response to see answer to reviewer #1, major point #4, for justification of this threshold). In contrast, for the variant at chr6:26090951:C:G (panel 22), there is a clear absence of IBD peak, indicating that carriers do not share IBD around this variant. Note that this variant is provided here as an example,

but it was removed from our analysis because of its frequency > 2%. Panel 25 illustrates the low IBD sharing for the variant chr7:117559590:ATCT:A, which was not classified as a founder variant based on this criterion (as also noted in our response to Reviewer #1, major point #4).

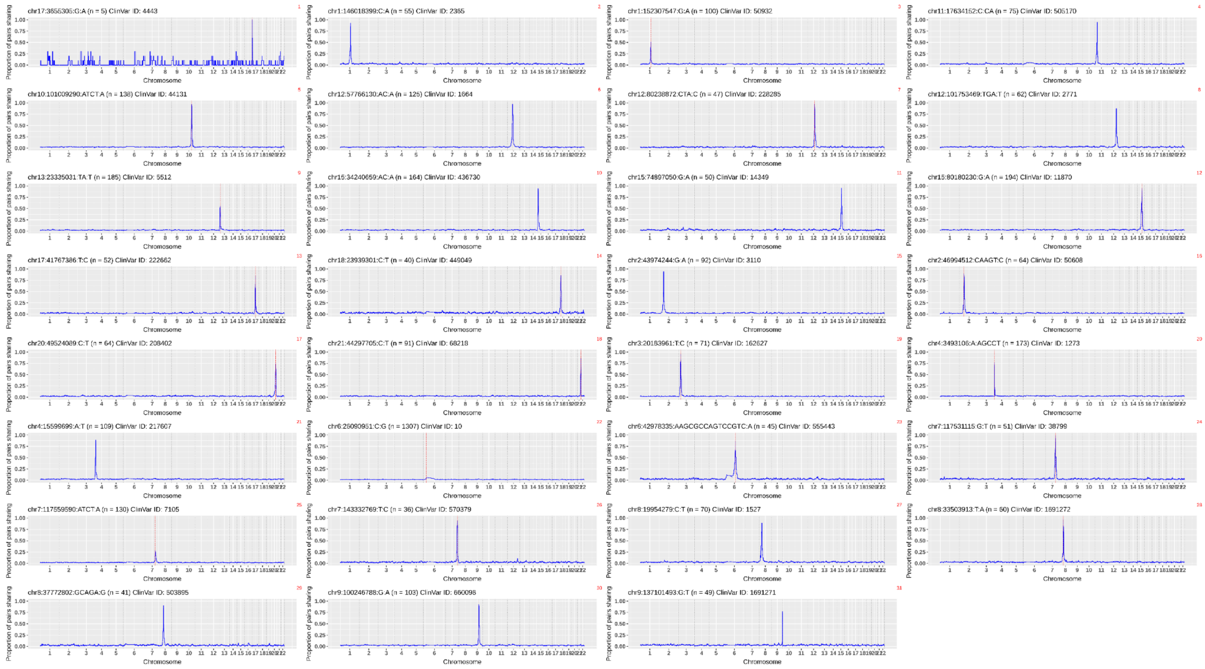

Figure 5: Proportion of IBD sharing graphs for some variants

• Lines 155-156: please specify the meaning of the numbers in parenthesis.
This was updated
• Please add to Table 3 the disease associated with each variant.
This was done, and the table was updated with the most up-to-date results from the clinic
• Lines 171-176: we expect that by pure luck, some previously discovered variants will not be discovered in the current study above a certain frequency. The probability for this can be computed using binomial distributions.
The reviewer is right and this was removed from the manuscript.
• Lines 182-186: similar work has been performed in other populations. See, for example, https://www.nature.com/articles/s41436-019-0676-x.
This reference was added in the text, line 196.
• Lines 190-191: there is a very long literature on the question of whether founder populations have an increased load of deleterious variants. It is not obviously true, because, while founder events increase the frequency of some variants, they eliminate all the variants in people not surviving the founder event. In particular, see https://www.nature.com/articles/ng.2896 and the reviews https://www.sciencedirect.com/science/article/abs/pii/S0959437X14001002 and https://www.nature.com/articles/nrg3931. There were also later papers.
This has been added in the text, line 214-216.
• Lines 193/245: I think that “fasten” and “fastening” are not used correctly.
This has been corrected, thanks for noticing.
• Supplementary figure 2: it is difficult to see how many variants are at each frequency given that points are overlapping.

We added total numbers for each category in the legend to make this more obvious.
• Lines 222-223: maybe I missed it, but I didn't see anywhere mentioned filtering by 10% false positives.
This was added in the supplementary table 5.
• Line 267: could you please provide details on the in-house Quebec reference panel?
This data is not published yet, a preprint was added as reference, line 289.
• Line 279: what were the parameters for removal of variants in LD?
This was added in the text.
• Line 294: how many samples were both the WGS dataset and in the imputed dataset? How many were only in the chip and are under a risk of misclassification?
All 1,852 WGS were in the imputed data, this was added in the text, line 316.
• Line 295: the possibility of a mix-up is unclear to me – assuming these are samples with both WGS and chip data, why not just compare the data between the platforms and verify it's the same person?
The reviewer is right that this is not due to mix-up, but rather samples that are at the edge of the clusters. This was corrected.
• Line 325: the carrier rate is just f_(hetero). Not the inverse.
This was changed in the text.
• Line 358: which variants were validated using the TaqMan assay?
This section was moved in the supplementary along with supplementary table 3. We added more information in the supplementary text.

References

1. Bchetnia, M. *et al.* Genetic burden linked to founder effects in Saguenay-Lac-Saint-Jean illustrates the importance of genetic screening test availability. *J Med Genet* **58**, 653–665 (2021).
2. Cruz Marino, T. *et al.* Portrait of autosomal recessive diseases in the French-Canadian founder population of Saguenay-Lac-Saint-Jean. *Am J Med Genet A* **191**, 1145–1163 (2023).
3. Laberge, A. M. *et al.* Population history and its impact on medical genetics in Quebec. *Clin Genet* (2005) doi:10.1111/j.1399-0004.2005.00497.x.
4. Scriver, C. R. Human Genetics : Lessons from Quebec Populations. *Annual Review of Genomics and Human Genetics* (2001) doi:10.1146/annurev.genom.2.1.69.
5. Isshiki, M. *et al.* Genetic disease risks of under-represented founder populations in New York City. *medRxiv* 2024.09.27.24314513 (2024) doi:10.1101/2024.09.27.24314513.
6. Garcia, A. M. *et al.* Using the ancestral recombination graph to study the history of rare variants in founder populations. 2025.03.13.643149 Preprint at

https://doi.org/10.1101/2025.03.13.643149 (2025).

7. Marafi, D. Founder mutations and rare disease in the Arab world. *Dis Model Mech* **17**, dmm050715 (2024).
8. Barashkov, N. A. *et al.* Autosomal recessive cataract (CTRCT18) in the Yakut population isolate of Eastern Siberia: a novel founder variant in the FYCO1 gene. *Eur J Hum Genet* **29**, 965–976 (2021).
9. Leckie, J. N. *et al.* Highly Elevated Prevalence of Spinobulbar Muscular Atrophy in Indigenous Communities in Canada Due to a Founder Effect. *Neurol Genet* **7**, e607 (2021).
10. Méndez, I. *et al.* Founder mutation in myosin-binding protein C with an early onset and a high penetrance in males. *Open Heart* **8**, e001789 (2021).
11. Ma, Y. *et al.* CLCC1 c. 75C>A Mutation in Pakistani Derived Retinitis Pigmentosa Families Likely Originated With a Single Founder Mutation 2,000-5,000 Years Ago. *Front Genet* **13**, 804924 (2022).
12. Yotova, V. *et al.* Anatomy of a founder effect: myotonic dystrophy in Northeastern Quebec. *Hum Genet* **117**, 177–187 (2005).
13. Fortin, C.-A. *et al.* Successes of an innovative population-based carrier screening program for four prevalent recessive hereditary diseases in a population with a founder effect in Quebec, Canada. *Genetics in Medicine Open* **0**, (2025).
