# Supplementary material for: Rare diseases load through the study of a regional population
Source: PLoS Genet. 2025 Oct 16;21(10):e1011876. doi: 10.1371/journal.pgen.1011876 (PMC12530595; doi:10.1371/journal.pgen.1011876)
Supplement: Attachment — Submitted filename: Michel_2025_rebuttal_2.pdf [file pgen.1011876.s005.pdf]

We thank the reviewers for their new reading of our revised manuscript. We are happy to report that we were able to address all of the remaining comments and we provide here a point-by-point review to each specific point that was raised.

Reviewer #1 minor comments:

I have only two minor suggestions that would further enhance the manuscript's clarity, though these are very minor and so do not warrant another round of review:

1. The sentence "Based on the absence of a clear definition for founder variants, we propose here a new definition of a founder variant" would benefit from briefly mentioning what that definition is within the same sentence or immediately following it.

We thank the reviewer for this suggestion. This was added at lines 126-129.

2. The section "Experimental validation of carrier rates" appears to interrupt the flow between figures and might be better positioned in the Supplementary Information to improve the manuscript's organization and readability.

The supplementary was reorganised.

Reviewer #2 partially or not addressed comments:

3. Reviewer assessment: Partially addressed. The authors are correct to observe that differences in mutation load are not primarily explained by differences in RFD or fold-difference, but rather differences in variant frequency. I am satisfied that the differences in variant frequency (or CR) are adequately described later in the manuscript, primarily in Figure 4. However, it would still be good to somehow quantify the observation on lines 104-105, which cites Figure 1. For instance, something like, “of the variants shown in Figure 1, __% had a frequency above X in QcP, whereas __% had a frequency above X in SLSJ.”

We acknowledge that the RFD>=10% was not well justified and presented. Thus, as suggested by the reviewer, we chose to remove this filtering step (except for the definition of founder variants, more on that in the next section). The reviewer’s suggestion was quite relevant since it doesn’t affect the results of our article and thus, was unnecessary.
Figure 1 has been redone with all variants instead of being restricted only to variants having an RFD>=10% following the reviewer’s suggestion (see comment #4). Numbers of variants were added at lines 96-99 as requested.

4. Reviewer assessment: Not addressed (rationale provided).
10% RFD: The authors have explained that the 10% RFD threshold was used to narrow down the ~200,000 ClinVar P/LP variants, and that including the variants with RFD < 10% would not change their observations about mutation load substantially, because most of them were rare, and/or not present in the imputed data. The “~200,000 ClinVar pathogenic variants” is misleading, however; the current version of Supplementary Figure 2 shows there are ~2,000 total ClinVar P/LP variants present in both QcP and gnomAD—I assume there are more found in just QcP but not gnomAD. It would be more accurate to say that the RFD

threshold was used to narrow down to 1,537 (later 1,302 in the imputed data) the initial set of ~2,000 ClinVar P/LP variants found in QcP and gnomAD, rather than the ~200,000 P/LP variants reported in ClinVar. Furthermore, at the allele frequencies relevant here, an RFD of 10% corresponds to very small absolute differences that may be indistinguishable from sampling noise at the QcP and gnomAD sample sizes (see minor point #16 below). The RFD threshold is therefore confusing and difficult to justify, both as a preliminary filter before looking for founder variants, and for observations of mutation load beyond CR/IBD-defined founder variants, as in Figures 1 and 2. Given that the RFD threshold seems arbitrary to analyses of mutation load (the authors have explained that it would not affect their observations about load in SLSJ vs UQc), I would still suggest that these analyses be simply repeated on the entire set of P/LP variants, regardless of RFD, primarily for the sake of conceptual clarity. Otherwise, the authors should clearly explain in the manuscript why an initial RFD >= 10% threshold was necessary, as opposed to simply looking at all rare P/LP variants in QcP, citing for instance counts and MAFs of ClinVar P/LP variants observed in QcP before and after the filter.

Figures 1 and 2 were redone to include all imputed variants instead of only those with RFD >= 10%.
For figure 1, all imputed variants present either in the QcP or in gnomAD were included and we still find multiple variants at a high frequency in the SLSJ. However, in contrast to the findings in Supplementary Figure 2, it indicates that high-frequency variants are more common in gnomAD than in the QcP. . Because it presented essentially the same information based on the WGS rather than imputed data, we removed Supplementary Figure 2. This change explains why we observed fewer variants for gnomAD, as we excluded all variants with frequencies below the smallest detectable in our WGS dataset (1/2037). Since the comparison between gnomAD and the QcP was not the scope of the present study, we didn’t specifically discuss this.
As for Figure 2, the results remain essentially unchanged after including all imputed variants instead of only those with an RFD ≥ 10%.
We acknowledge that the RFD threshold is arbitrary with respect to mutation load analysis, which is why we now present those results without applying the threshold. Nevertheless, we chose to keep the RFD≥10% criteria for defining founder variants. As stated in the manuscript, there is no universally accepted definition of a founder variant in the literature. According to the NIH (https://www.cancer.gov/publications/dictionaries/genetics-dictionary/def/founder-mutation), a founder variant is:

> ‘A **genetic alteration observed with high frequency** in a group that is or was geographically or culturally isolated, in which one or more of the ancestors was a carrier of the altered gene. This phenomenon is often called a founder effect.’

In practice, to consider a variant as founder, most studies rely primarily on its frequency within the ‘source’ and ‘founder’ populations, as seen in the four reviews cited in our manuscript[1–4] and in more recent publications[5–7]. In line with these publications and the NIH definition, we consider the use for a higher frequency threshold justified.

ClinVar 1* vs 2* Review Status: The authors have explained that they did not repeat their analyses of mutation load in Figure 2 (or Figure 5) using only P/LP variants with review status >= 2*, because some 1* variants might be clinically relevant, including 11 of the previously-documented variants discussed in the study, and a newly-identified founder

variant which was observed alongside the relevant phenotype. Because the aim of their study was to identify variants which might be linked to a founder disease, they did not find it necessary to restrict to >= 2* in any of their analyses. I agree that this rationale makes sense for identifying potentially pathogenic founder variants, however, another purpose of the study (included in its title) is to compare the mutation load of these variants between SLSJ and UQc. It would be informative and straightforward to add a simple sensitivity panel to Figs 2 and 5 restricted to ClinVar >=2* (plus previously documented founders) to show whether the trends persist when limited to variants with stronger evidence of pathogenicity. I also note that the term “mutation load” formally relates to fitness; if you keep this terminology, please define it operationally in terms of your potentially pathogenic count-based proxy and acknowledge limits (penetrance/inheritance/varying evidence of pathogenicity).
Small panels were added for figures 2 and 5 showing results restricted to variants with a ClinVar review status of at least two stars. The trends remain unchanged, so our conclusions on mutation load also hold when considering only variants with stronger evidence of pathogenicity.
In response to the reviewer’s comment regarding terminology, we have defined the term “mutation load” in the manuscript at line 222.

5. Reviewer assessment: Partially addressed. The authors have adequately clarified in the supplementary text that the additional 1,000 SLSJ individuals come from an independent cohort and are not a replication of CARTaGENE. The validation of the nine carrier rates is still referenced in lines 120-121; it would be good to very briefly add some clarifying wording here, e.g. “nine carrier rates were assessed in an independent cohort of 1,000 SLSJ individuals in the CIUSSS laboratory, and…” so that readers don’t need to go to the supplemental material to understand this.
This was changed at line 123.

Minor Points:
6. Reviewer assessment: Partially addressed. I appreciate the authors specifying they are concerned with rare Mendelian diseases. In following up on the edit made to line 60, however, I was unable to find any count of more than 10,000 rare Mendelian diseases in Orphanet, let alone rare Mendelian diseases. At the URL https://www.orpha.net/ as of July 2025 there are 6528 diseases under “Orphanet in numbers”, not all of which are necessarily genetic or Mendelian. The authors’ citation in the previous sentence (line 59, citation 1) is to Haendel et al. 2020, which estimates there are "more than 10,000" rare diseases (not necessarily all genetic or Mendelian), but this was using Mondo, which draws upon Orphanet and other resources like OMIM, rather than just within Orphanet. There is a count of Mendelian human diseases in Mondo at the URL https://mondo.monarchinitiative.org/ under “Representation of Disease Types”, which is at 11,566 for Mendelian human diseases as of July 2025. The authors should clarify this and add an appropriate citation.
This was changed at line 59, the Orphanet site was updated after we submitted the present study.

16. Reviewer assessment: Partially addressed. I appreciate the authors having added some conversions between RFD and fold-difference to the methods section. The authors use the RFD >= 10% threshold to indicate that variants are more common in QcP than in gnomAD v3.1.2 NFE genomes. There are 29,353 individuals in the imputed QcP dataset, and I believe there are something like ~33,000 individuals in the gnomAD v3.1.2 NFE genomes. At

very low frequencies, an RFD of 10% corresponds to very small absolute differences (e.g., AF 0.005 vs 0.0045 at CR ~1/200). Would a 10% RFD for a variant with CR=1/200 in QcP be distinguishable from sampling noise for the QcP and gnomAD sample sizes? The authors should comment on this in the RFD Methods section.

This was added at lines 354-355.

References


1. Bchetnia, M. *et al.* Genetic burden linked to founder effects in Saguenay-Lac-Saint-Jean illustrates the importance of genetic screening test availability. *J Med Genet* **58**, 653–665 (2021).
2. Cruz Marino, T. *et al.* Portrait of autosomal recessive diseases in the French-Canadian founder population of Saguenay-Lac-Saint-Jean. *Am J Med Genet A* **191**, 1145–1163 (2023).
3. Laberge, A. M. *et al.* Population history and its impact on medical genetics in Quebec. *Clin Genet* (2005) doi:10.1111/j.1399-0004.2005.00497.x.
4. Scriver, C. R. Human Genetics : Lessons from Quebec Populations. *Annual Review of Genomics and Human Genetics* (2001) doi:10.1146/annurev.genom.2.1.69.
5. Isshiki, M. *et al.* Genetic disease risks of under-represented founder populations in New York City. *medRxiv* 2024.09.27.24314513 (2024) doi:10.1101/2024.09.27.24314513.
6. Garcia, A. M. *et al.* Using the ancestral recombination graph to study the history of rare variants in founder populations. 2025.03.13.643149 Preprint at https://doi.org/10.1101/2025.03.13.643149 (2025).
7. Marafi, D. Founder mutations and rare disease in the Arab world. *Dis Model Mech* **17**, dmm050715 (2024).
